# Case Report: Epstein-Barr virus negative lymphoepithelioma-like cholangiocarcinoma: a rare tumor that deserves further exploration. Report of a case with distinct genomic and clinical features

**DOI:** 10.3389/fonc.2025.1620328

**Published:** 2025-08-04

**Authors:** Salvatore Corallo, Chiara Milani, Alessandro Vanoli, Anna Gallotti, Anna Pagani, Francesco Serra, Marcello Maestri, Valentina Ravetta, Angioletta Lasagna, Paolo Pedrazzoli, Francesco Agustoni

**Affiliations:** ^1^ Department of Internal Medicine and Medical Therapy, University of Pavia, Pavia, Italy; ^2^ Unit of Oncology, Fondazione IRCCS Policlinico San Matteo, Pavia, Italy; ^3^ Department of Molecular Medicine, University of Pavia, Pavia, Italy; ^4^ Unit of Anatomic Pathology, Fondazione IRCCS Policlinico San Matteo, Pavia, Italy; ^5^ Institute of Radiology, Fondazione IRCCS Policlinico San Matteo, Pavia, Italy; ^6^ Division of General Surgery 1, Fondazione IRCCS Policlinico San Matteo, Pavia, Italy; ^7^ Gastroenterology and Endoscopy Unit, Fondazione IRCCS Policlinico San Matteo, Pavia, Italy

**Keywords:** lymphoepithelioma-like cholangiocarcinoma, HCC-CC double primary tumor, intracolonic metastasis, EBV-negative cholangiocarcinoma, HCV-related cirrhosis

## Abstract

Lymphoepithelioma-like cholangiocarcinoma (LEL-CC) is a rare variant of cholangiocarcinoma characterized by undifferentiated malignant epithelial cells and a significant lymphoid infiltrate. Due to its rarity, there is currently no established treatment protocol for LEL-CC, and limited data are available regarding the genomic landscape of this rare tumor. In this report, we present the case of an 84-year-old woman with a history of Hepatitis C-related cirrhosis who was diagnosed with EBV-negative LEL-CC. This tumor presented as a double primary tumor together with a moderately differentiated hepatocellular carcinoma, at the time of first diagnosis. After surgical resection, the patient experienced a relapse with intracolonic metastasis. Comprehensive genomic profiling revealed unique genetic features consistent with LEL carcinoma of other sites of origin. The genetic and clinical characteristics of our case highlight the need for further research on this rare variant of cholangiocarcinoma. Gaining insights into the molecular mechanisms behind this type of cancer could lead to the development of effective targeted therapies or immunotherapeutic approaches.

## 1 Introduction

Lymphoepithelioma-like cholangiocarcinoma (LEL-CC) is a rare variant of cholangiocarcinoma (CC) characterized histologically by lymphoid cells infiltrating the tumor and the surrounding stroma. The first case of LEL-CC was reported by Hsu et al. in 1996 (1). Since then, additional cases have been documented, and the number of reports has increased in recent years (2).

Tumors exhibiting lymphoepithelioma-like features have been reported in various organs, including the gastrointestinal tract, lungs, salivary glands, thymus, and urinary tract (3–6). Similar to the more widely recognised lymphoepithelioma-like variant of gastric cancer (7), a connection between Epstein-Barr virus (EBV) infection and this uncommon variant of CC has been extensively reported (8). Some evidence suggests that EBV infection may be directly implicated in the tumorigenesis of LEL-CC (9). However, not all EBV-associated CC cases exhibit lymphoepithelioma-like features, and approximately 30% of LEL-CCs are found to be EBV-negative (8).

LEL-CC often lacks specific symptoms, making it frequently incidentally detected in asymptomatic patients during imaging studies. The studies that have examined the radiological features of LEL-CC showed that, compared to classical intrahepatic cholangiocarcinomas (iCCs), LEL-CCs appear as masses with a combination of hypervascularity, washout, delayed intratumoral enhancement, or pseudocapsule enhancement, and a well-defined boundary (10, 11). However, these imaging features can overlap with those of metastases, iCC, and hepatocellular carcinoma (HCC) (12), which means the diagnosis of LEL-CC primarily depends on histopathology.

The histopathological diagnosis of LEC-CC relies on identifying poorly differentiated, large polygonal tumor cells with vesicular nuclei and prominent nucleoli, arranged in nests, sheets, or cords, and on the presence of a prominent lymphoid stroma, which is often densely infiltrated by both T and B lymphocytes, occasionally forming lymphoid follicles. The immunohistochemical profile of LEC-CC is characterized by positive staining for biliary epithelial markers and negative expression of hepatocellular markers (13, 14). These histological features, which resemble those of nasopharyngeal carcinoma, help differentiate LEC-CC from other primary liver carcinomas. However, the specific amount or density of lymphocyte infiltration required for diagnosis has not yet been established, while mixed patterns including well-to-moderately differentiated glandular components have been described (15).

Although LEL-CC is considered a rare disease, an increasing number of cases have recently been reported (2, 16). However, there is still limited knowledge regarding the molecular characteristics of this tumor and the best treatment strategies for advanced disease. Here, we present a case of an extensively genomically characterized EBV-negative LEL-CC that presented as a double primary tumor with HCC and exhibited atypical metastatic spread to the colon at the time of the first relapse after surgery.

## 2 Case description

**Figure 1:**
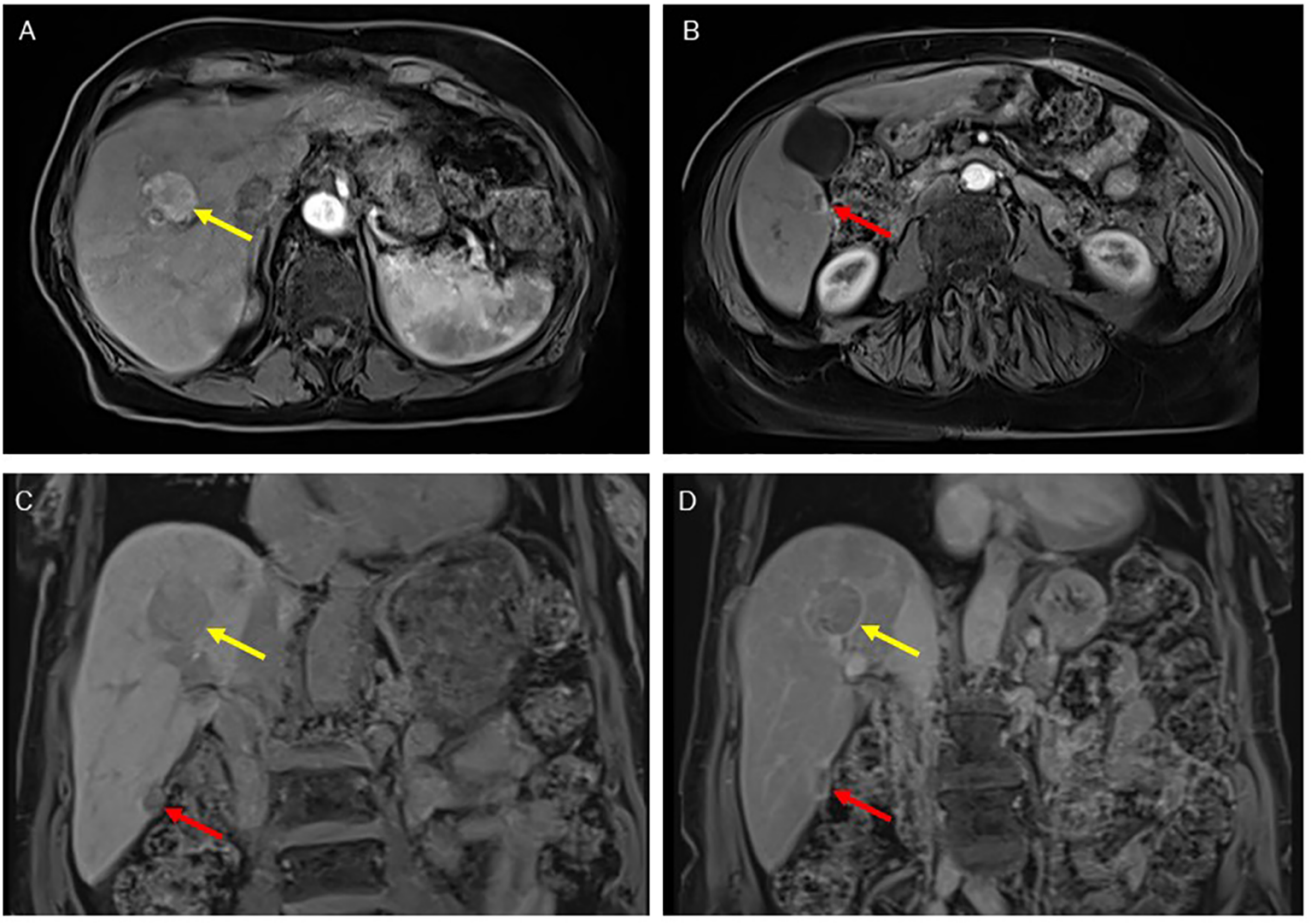
MR images at diagnosis. The arterial phase enables the differential diagnosis between the lesion in the VIII segment (yellow arrow), which is homogeneously hypervascularised as typical for HCC **(A)**, and the lesion in the VI segment (red arrow), characterized by a rim of enhancement typical of CC **(B)**. In the venous phase, both lesions showed a clear washout, with a demarcated pseudo-capsule for the HCC **(C)**. In the hepatobiliary phase, both were hypointense **(D)**.

An 84-year-old female presented in October 2019 to the outpatient department of Internal Medicine at the Fondazione IRCCS Policlinico San Matteo in Pavia for follow-up of cirrhosis. She was diagnosed with hepatitis C virus (HCV) infection in 1998 and received treatment with Peg-Interferon Alfa and Ribavirin. However, both treatments were discontinued in 2006 due to side effects, and she did not achieve viral eradication. She had two hepatic nodules identified on ultrasound: one in segment VIII, measuring 37 mm, and another in segment VI, measuring 14 mm. The abdominal Magnetic Resonance Imaging (MRI) confirmed the presence of two nodules, each exhibiting distinct radiological features. The lesion in segment VIII displayed a well-defined margin resembling a pseudo-capsule, along with homogeneous marked enhancement in the arterial phase, with clear wash-out in the portal-venous phase, where the pseudo-capsule remained well-defined, as usually noted in HCC (**Figures 1A, C**). In contrast, the lesion in segment VI showed signs of liver capsule retraction and a peripheral rim of arterial enhancement that persisted in the venous phase, which is characteristic of CC (**Figure 1B**). Both lesions appeared hypointense in the hepatobiliary phase (**Figure 1D**). Laboratory tests showed good liver function. The blood tumor markers carcinoembryonic antigen (CEA) and Ca 19.9 were both within normal ranges, and the blood alpha-fetoprotein (AFP) level was 11554.8 IU/ml. Additionally, the markers for hepatitis B virus (HBV) infection, including the hepatitis B surface antigen and core antigen, were negative.

An ultrasound-guided fine needle biopsy of both nodules was performed, and the pathological report indicated the presence of moderately differentiated HCC at the VIII segment, expressing Hep-Par1 and glutamine synthetase, and with a partial positivity for AFP and glypican 3. On the contrary, the pathological report of the nodule in the VI segment revealed a poorly differentiated carcinoma, with immunohistochemical characteristics that excluded a hepatocellular differentiation (expression of CK8/18, CK7, and CK19 and negativity for glypican3, AFP, Hep-Par1, CDX2, TTF1, PAX8, p40, and synaptophysin).

Initially reluctant to undergo surgery, the patient received multiple percutaneous ethanol injections between December 2019 and February 2020, resulting in a favorable response for the nodule in the segment VIII, but noted the growth of the VI segment nodule, as revealed by a CT computed tomography (CT) scan in August 2020 which showed an increase in the size of the nodule in the VI segment, measuring 30 x 39 mm with a necrotic central portion, along with an inhomogeneous border characterized by intense and homogeneous contrast enhancement. The blood tumor markers, CEA and Ca 19.9, remained within normal ranges, but the blood AFP level was elevated at 347.6 IU/ml.

**Figure 2:**
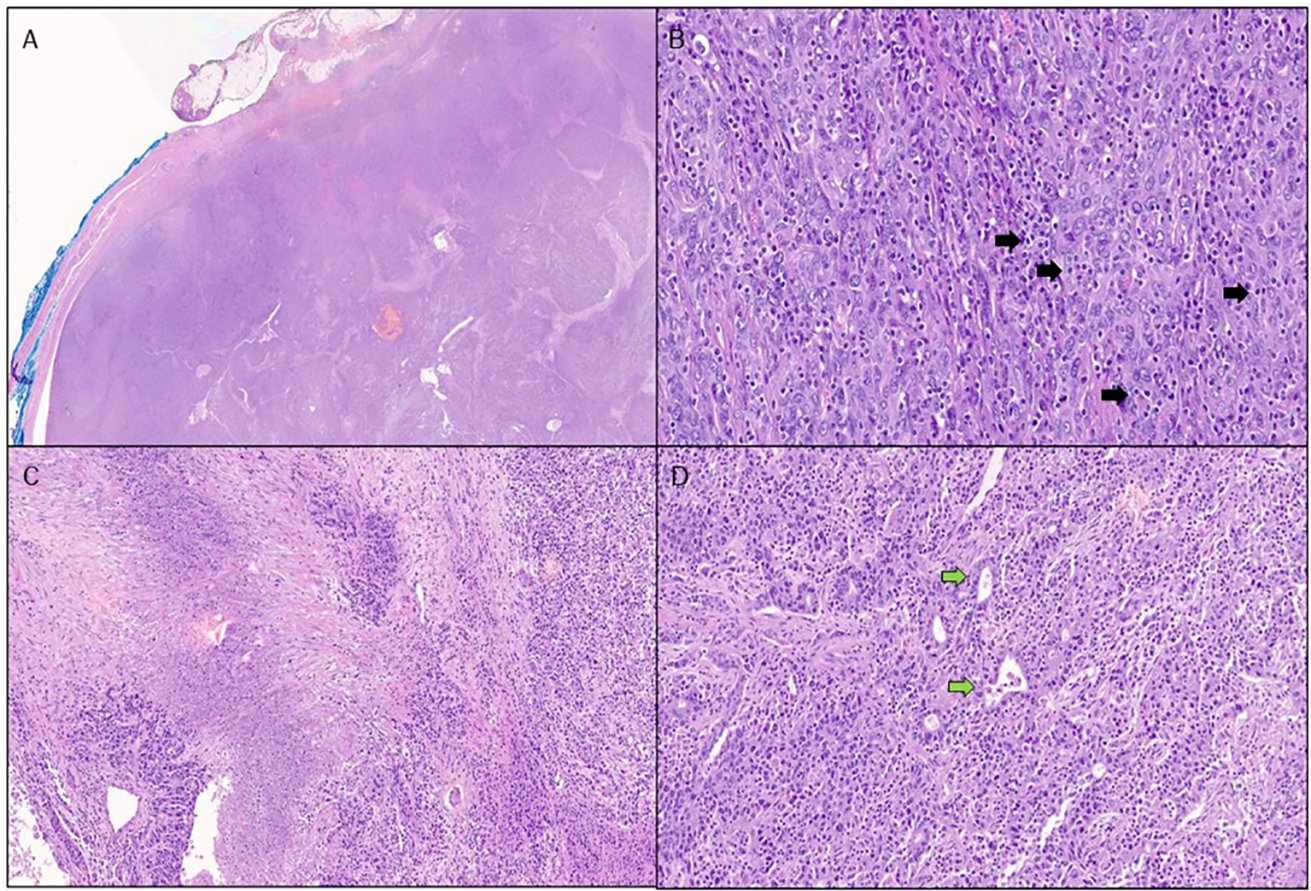
Pathological features of our reported case of lymphoepithelioma-like cholangiocarcinoma. Stained with hematoxylin and eosin: at low power, the nodule shows predominantly pushing borders **(A)**. At higher power, the neoplasm is poorly differentiated, with a solid architecture, and is composed of tumor cells exhibiting a syncytial appearance and vesicular nuclei. Many tumor-infiltrating lymphocytes are visible (black arrows), both within tumor nests and in the surrounding stroma **(B)**. Tumor necrotic areas are easily appreciated. **(C)** Areas with glandular differentiation (green arrows) are focally present **(D)**.

After a thorough discussion by the multidisciplinary team, in September 2020, the patient underwent a laparotomic tumor resection of the nodule in the VI segment. The pathology report revealed an LEL-CC in the context of mixed type of cirrhosis (micro and macronodular). The tumor was described as a poorly differentiated neoplasia consisting of large, atypical, fusiform elements with vesicular nuclei, solid-syncytial growth patterns, and focal glandular structures. Associated features included areas of necrosis, stromal desmoplasia, and intense intralesional inflammatory infiltration characterized by numerous CD3+ T lymphocytes. The immunohistochemical profile was consistent with the diagnosis of LEL-CC (CK8/18+, EMA+, CK7 +/-, CK19 +/-, CK20 +/-, Hep-Par1 -) (**Figure 2**). *In situ* hybridisation (ISH) for EBV-encoded RNA (EBER) was negative. Immunohistochemical analysis showed a proficient mismatch repair profile (positive staining for MLH1, PMS2, MSH2, and MSH6). There was evidence of microvascular invasion in the liver tissue near the cancer, but no signs of nerve invasion. No post-operative chemotherapy was administered, and the patient continued her regular clinical and radiologic follow-up.

By February 2021, a new nodule in segment III (21 mm × 17 mm) was detected, with an AFP level of 475.5 IU/mL. After a new multidisciplinary discussion, the patient underwent a laparoscopic wedge resection of the III segment of the liver in May 2021. The pathology report confirmed the diagnosis of LEL-CC.

**Figure 3:**
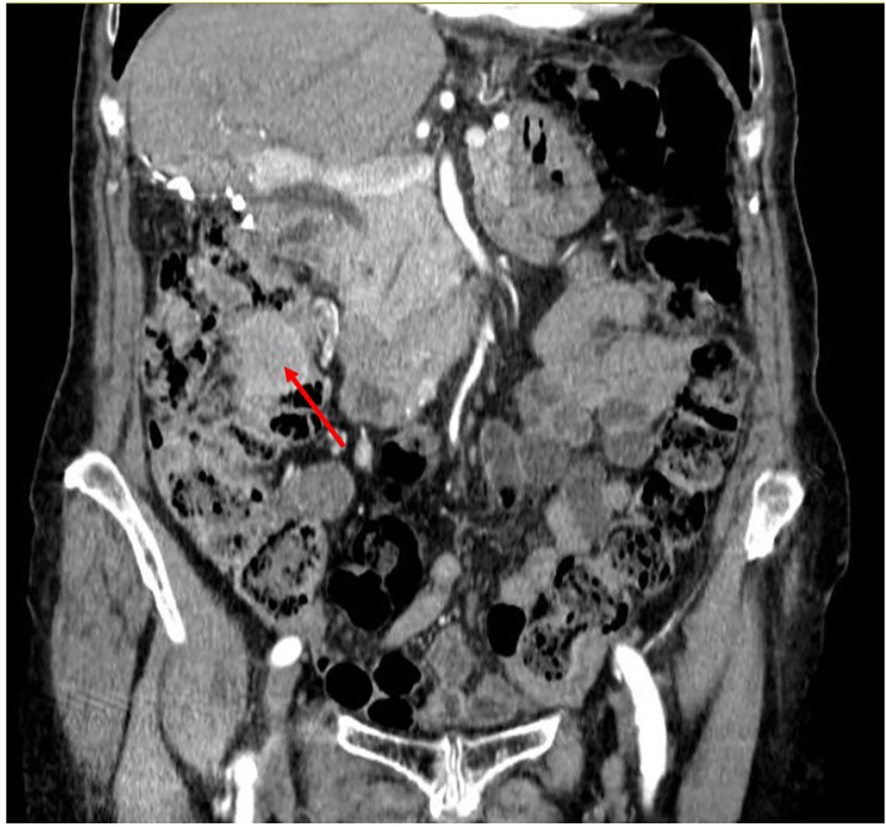
CT scan image showing the intracolonic metastasis. Coronal CT, in venous phase, shows a homogeneous mass in the ascending colon (red arrow) without abnormal upstrem dilatation.

**Figure 4:**
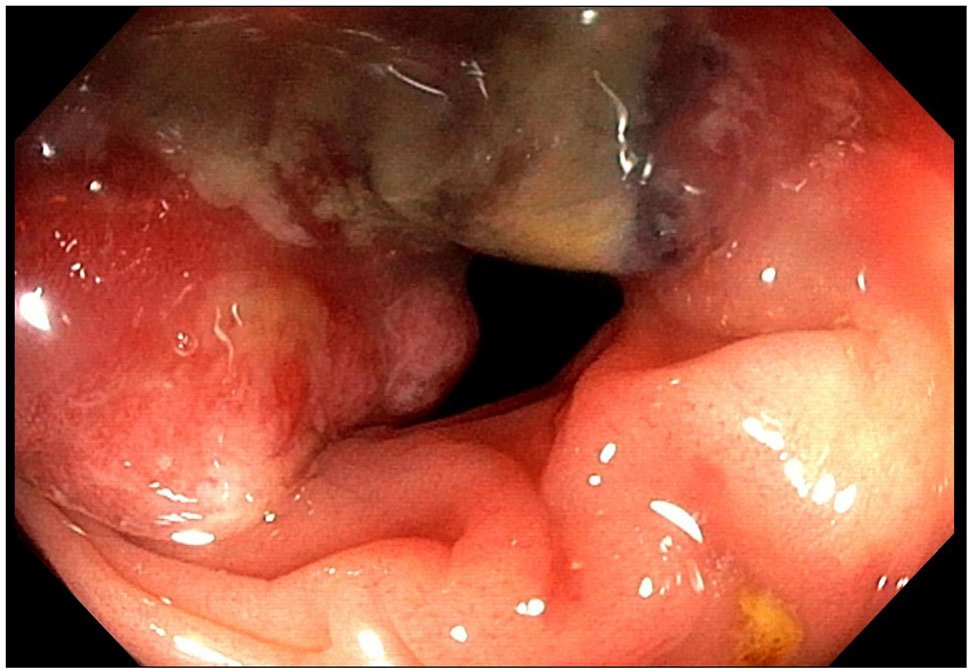
Colonoscopy showing a semi-circumferential, substenosing mass in the hepatic flexure of the colon.

The first follow-up CT scan evaluation, conducted in July 2021, showed the development of a new hepatic nodule in the VI segment. Additionally, there was a nodule measuring 4 x 3 cm located within the lumen of the ascending colon, which had not been detected in previous CT scans (**Figure 3**). A colonoscopy revealed a stenosing neoformation in the hepatic flexure of the colon, which was impassable with the standard colonoscope (**Figure 4**). An agobiopsy of the colon demonstrated poorly differentiated neoplasia with an immunohistochemical profile compatible with a metastasis from the recently resected LEL-CC. Due to the risk of colonic occlusion, the patient underwent a right hemicolectomy with a lateral ileo-colic anastomosis. The pathology report confirmed the diagnosis of metastasis from the previously resected LEL-CC.

From September 2021 to February 2022, the patient received seven cycles of postoperative chemotherapy with Cisplatin and Gemcitabine, achieving a partial response, with a 54% reduction in the sum of the diameters. Following a treatment break and subsequent disease progression, retreatment with Cisplatin and Gemcitabine resulted in a partial response, characterized by a 44% reduction in target lesion diameters. Considering the limited extent of the disease and the short progression-free interval during the previous treatment break, the patient underwent a wedge resection of the VI hepatic segment. The pathology report confirmed histological and immunohistochemical findings consistent with metastasis from the prior LEL-CC. Immunohistochemical staining for PD-L1 (**Supplementary Figure 1**) indicated high PD-L1 expression in both tumor and immune cells: tumor proportion score (TPS) was 50%; combined positive score (CPS) was 60 (17). A comprehensive genomic profiling of the surgical specimen, conducted as part of a clinical trial a clinical trial (NCT05918666) using the Foundation One CDx (F1CDx) assay, revealed the following genetic alterations: *MET*, *CCND1*, *FGF19*, *FGF3*, and *FGF4* amplification, *NFKBIA* mutation (W11fs*1), and a *TERT* promoter mutation (124C>T). The tumor mutation burden was 4 mutations per megabase.

The patient remained disease-free until February 2023, when new liver lesions prompted the initiation of Folfox-6, resulting in a partial response (58% reduction in target lesions). This new treatment regimen was preferred over re-treatment with Cisplatin and Gemcitabine due to the persistence of cumulative toxicity from Cisplatin (Grade 1 hypoacusia). However, the treatment was complicated by Grade 1 thrombocytopenia, Grade 2 paresthesia, and Grade 2 fatigue, leading to a therapy break after seven cycles.

In December 2023, a follow-up CT scan revealed disease progression in the liver and retroperitoneal lymph nodes. A new treatment using gemcitabine as a single agent was initiated, but, unfortunately, the first radiologic reassessment indicated disease progression in the liver. In April 2024, a new treatment regimen with capecitabine was initiated, resulting in stable disease, accompanied by a slight reduction in tumor size (a 21% decrease in the sum of the target lesions). This treatment continued until November 2024, when a CT scan of the abdomen revealed disease progression in the liver. Following a treatment break due to a temporary worsening of fatigue, a new treatment with FOLFIRI was started in March 2025, and it is still ongoing.

## 3 Discussion

To the best of our knowledge, this is the first report on an EBV-negative LEL-CC diagnosed as an incidental double primary tumor with an HCC and recurring with an atypical intracolonic metastasis.

The simultaneous occurrence of synchronous HCC and iCC in the same patient is a rare event, with an estimated prevalence of less than 0.5% (18, 19). Previous reports revealed that this rare condition is more common among patients with viral hepatitis or cirrhosis, which are established risk factors for both HCC and iCC (19–21). However, given that HCC is frequently diagnosed based solely on imaging findings, particularly in the past, the true prevalence of synchronous double primary may be higher than currently reported.

Previous studies suggest that the stage of the iCC primarily determines the prognosis of patients with HCC-iCC double primary and that it is significantly worse compared to HCC alone (18, 22). Therefore, misdiagnosing a double primary liver cancer may have important treatment implications.

In our case, the marked difference in radiologic features among the two nodules incidentally detected in the context of a cirrhotic liver suggested a biological difference between the two neoplastic lesions. Indeed, the radiologic features of LEL-CC generally resemble those of conventional iCC; however, some differences have been noted, such as a higher frequency of smooth margins, non-rim arterial phase hyperenhancement, absence of perilesional enhancement, and liver capsular retraction (23). Consequently, our case highlights the importance of accurate differential radiologic diagnosis and liver biopsy when multiple nodules are present in a cirrhotic liver, mainly when differences in radiologic features between the nodules are observed.

At the time of disease relapse following the first surgery, the patient presented with a colonic metastasis. Common sites for distant metastasis from iCC include the liver and distant lymph nodes, followed by the peritoneum, lungs, bones and brain (24, 25). Notably, intracolonic metastasis is a rare event; to our knowledge, only six cases (26–31) have been reported in the literature (**Supplementary Table 1**). The absence of signs indicating peritoneal seeding in these limited case reports suggests that the most likely mode of metastasis to the colon was through hematogenous spread. Consistent with this evidence, our case showed no signs of peritoneal metastases, despite a long follow-up period after the metastasectomy. Furthermore, the patient did not undergo trans-arterial chemoembolization, which could have led to the development of retrograde hematogenous metastases, thus supporting the hypothesis of a primary atypical hematogenous site of metastasis.

In terms of prognosis, our patient demonstrated a long-lasting response to multimodal treatment strategies comprising various chemotherapy regimens (**Supplementary Figure 2**). This approach resulted in an extraordinary overall survival rate from the time of diagnosis of metastatic disease. The prolonged survival of our patient, along with her high sensitivity to most of the chemotherapy regimens used in the metastatic setting, distinguishes her as an outlier compared to typical iCCs. Recent randomised controlled trials indicate that the median expectable overall survival for completely resected CC is approximately 36 to 50 months (32) and it decrease to 11 to 13 months in the metastatic setting (33). Notably, our patient is still alive approximately 65.7 months after the initial diagnosis of liver-limited disease and 44.7 months after the diagnosis of metastatic spread. Some studies have suggested that most patients with hepatic LEL-CC have a favourable prognosis, and reports on metastatic LEL-CC show median overall survival rates ranging from 13 to 100 months after diagnosis (34). A recent study by Huang YH et al., which retrospectively analysed 303 iCC cases, indicated that LEL-CC is more common in EBV-associated (EBVa) iCC (EBVa-iCC) and that EBV-positive LEL-CCs are associated with significantly higher survival rates compared to conventional EBVa-iCCs and non-EBVa-iCCs (35). Unfortunately, there is limited data available regarding the prognosis of EBV-negative LEL-CCs. Although EBV-associated malignancies in the stomach and lung are associated with better prognoses compared to EBV-negative cases (36–38), a recent study by Wang et al. on 13 cases of LEL-CC reported that the absence of EBV infection correlated with a better prognosis (39). However, due to the scarcity of available data, further evidence is needed to confirm the prognosis of LEL-CC compared to conventional iCC and to clarify the relationship between EBV infection status and prognosis.

In the present case, the negative results of EBER1/2 ISH confirm the absence of a relation to EBV infection. In contrast, our patient had a long history of HCV infection that failed to achieve virus eradication and had been diagnosed with cirrhosis. Previous studies (16, 40–43) have shown a low prevalence of HCV and cirrhosis among LEL-CCs, with rates of approximately 5-7% and 10-15%, respectively. Conversely, HBV infection has been reported in about 27-43% of LEL-CCs (16, 40–44), suggesting that HBV may play a role in the tumorigenesis of LEL-CC, similar to EBV. On the contrary, the prevalence of cirrhosis, along with HCV and HBV infection, is higher among lymphoepithelioma-like-HCC (LEL-HCC), ranging from 35 to 45% (8, 16) (**Table 1**). Furthermore, the vast majority of LEL-HCC cases are negative for EBV, which suggests that cirrhosis, rather than the underlying infection, may have a dominant role in the disease’s pathogenesis. In terms of underlying liver disease, our case is more similar to LEL-HCC than LEL-CC. Although a synchronous HCC was detected at the first diagnosis, the cholangiocarcinoma differentiation of our LEL-CC has been confirmed by the pathological reports of multiple surgical specimens obtained over time.

**Table 1:** Clinicopathological and molecular characteristics of EBV-positive and EBV-negative LEL-CC and LEL-HCC cases reported in the literature.

Features	EBV-positive LEL-CC (N=86)^1^	EBV-negative LEL-CC (N=24) ^1^	LEL-CC Overall (N=110) ^1^	LEL-HCC Overall (N=57)^2^
Sex				
Female Male	61 (70.9) 25 (29.1)	8 (33.3) 16 (66.7)	69 (62.7) 36 (37.3)	21 (36.8) 36 (63.2)
Age				
Mean ± SD	52.75 ± 12.6	61.21 **±** 10.01	54.6 **±** 12.5	58.9 **±** 16.4
Tumor Location				
Right lobe Left lobe NR	37 (43) 39 (45.4) 10 (11.6)	14 (58.3) 8 (33.3) 2 (8.3)	51 (46.4) 47 (42.7) 12 (10.9)	6 (10.5) 4 (7.0) 46 (80.7)
Tumor size, mean (±SD) mm				
	42.96 ± 26.54	33.54 ± 17.14	40.89 **±** 25.02	37.8 **±** 24.3
HBV infection				
Yes No NR	31 (36.0) 51 (59.3) 4 (4.7)	14 (85.3) 10 (41.7) -	45 (40.9) 61 (55.5) 4 (3.6)	25 (43.9) 32 (56.1) -
HCV infection				
Yes No NR	1 (1.2) 73 (84.9) 12 (14.0)	5 (20.8) 18 (75.0) 1 (4.2)	6 (5.5) 91 (82.7) 13 (11.8)	19 (33.3) 38 (66.7) -
Chirrosis				
	5 (5.8)	8 (33.3)	13 (11.8)	25 (43.9)
Predominant histology				
Undifferentiated pattern Glandular pattern Both NR	21 (24.5) 34 (39.5) 7 (8.1) 24 (27.9)	20 (83.3) 1 (4.2) 2 (8.3) 1 (4.2)	41 (37.3) 35 (31.8) 9 (8.2) 25 (22.7)	NA
Genomic features	N=30 (%)^3^	N=10 (%)^3^	N=40 (%)^3^	N=12 (%)^4^
*pTERT* mutation *TP53* mutation *KRAS* mutation *NRAS* mutation *ARID1B* mutation *POLE* mutation	3 (10) 1 (3.3) 0 1 (3.3) 1 (3.3) 1 (3.3)	6 (60%) 7 (70%) 1 (10%) 0 0 0	9 (22.5) 1 (2.5) 1 (2.5) 1 (2.5) 1 (2.5) 1 (2.5)	0 1 (8.3) 0 0 0 0

NR, not reported; SD, standard deviation.

^1^Based on data from Tsai et al. (15), Zheng et al. (42), Li et al. (43), Zhang et al. (16).

^2^Based on data from Lagbaa et al. (8), and Chan et al. (45).

^3^Based on data from Tsai et al. (15), Zheng et al. (42).

^4^Based on data from Chan et al. (45).

Interestingly, the genomic profile of our case *(MET*, *CCND1*, *FGF19*, *FGF3*, and *FGF4* amplification, *NFKBIA*, and *TERT* promoter mutation) resembles both the molecular landscape of LEL-HCC and LEL-CC. A recent analysis by Tsai et al. reported that mutations of *TERT* and *TP53* are the most frequently detected mutations in LEL-CC, whereas gene alterations typically associated with cholangiocarcinoma, such as *IDH1*, *IDH2*, *ARID1A*, *ARID2*, and *BAP1* mutations, as well as *FGFR2* fusions, have not been described in LEL-CC (15).

On the other hand, as recently reported by Chan et al. (45), approximately 25% of LEL-HCC cases harbour a focal amplification at 11q13.3, a locus that contains *CCND1*, *FGF19*, and *FGF4.* This gene alteration is minimally detected in conventional HCC. The strong relationship between genomic profile and histopathological phenotype may suggest that transcriptomic products of these genes may play a role in carcinogenesis, potentially providing a proliferative advantage to tumor cells. We could hypothesise that these gene alterations might also lead to the high immune recognition typically described in LEL-CC carcinomas and the activation of immune checkpoint inhibitory signals. Supporting this hypothesis, data from The Cancer Genome Atlas (TCGA) HCC dataset indicate that a subgroup of HCC with high expression of checkpoint genes has a high frequency of amplification of *CCND1, FGF19, FGF4*, and *MET*, which are located on the locus 11q13.3 (45).

Our case exhibited extremely high PD-L1 expression, suggesting that the molecular mechanisms associated with immune evasion may be linked to the activation of inhibitory immune checkpoints. Recent data indicate that approximately 50-75% of LEL-CC demonstrate higher PD-L1 expression compared to conventional iCC (15, 39). Based on the high PD-L1 levels reported in EBV-infected nasopharyngeal carcinoma (NPC) (46), EBV-related B-cell lymphoma, EBV-associated gastric cancers (7), and EBV-positive Hodgkin lymphoma (47), it can be argued that the chronic inflammatory environment of EBV-associated cancers may promote the upregulation of PD-L1 and the PD-L1/PD-1 axis (39). Intriguingly, data from the TGCA gastric cancer dataset (48) show that EBV-positive gastric cancer frequently exhibits amplification at 9p24.1, a locus containing *CD274* and *PDCD1LG2* (encoding PD-L1 and PD-L2), which is associated with a more immune-active profile. The prevalence of high PD-L1 protein expression is also significantly greater in LEL-HCC compared to conventional HCC (about 65% versus 6%) (45), even though EBV is primarily negative in LEL-HCC. These data further suggest that in EBV-negative LEL carcinomas, genomic alterations may guide both immune recruitment and immune evasion. Conversely, in EBV-positive LEL carcinomas, these alterations are likely driven primarily by genomic and epigenetic changes associated with EBV infection.

The combination of high immune infiltration and elevated PD-L1 expression in LEL carcinomas suggests that LEL features could serve as a potential marker for benefiting from anti-PD-1/PD-L1 therapies. Accordingly, previous studies have demonstrated that immune checkpoint inhibitors (ICIs) are effective against pulmonary LELC with high PD-L1 expression (49–52). Unfortunately, evidence regarding the efficacy of immunotherapy in LEL-CC is scarce, although some reports indicate encouraging results (53–56). Regrettably, our patient did not receive immunotherapy because ICIs have only recently been approved and reimbursed in Italy for the treatment of CC, with restrictions limiting access to untreated advanced patients or those with high microsatellite instability (MSI-high) status.

In conclusion, the atypical clinical and genomic profiling of our case highlights that LEL-CC should be recognized as a distinct type of primary liver cancer. This condition warrants further investigation to better understand its genomic and immunologic landscape, which could lead to the development of effective, customized treatment strategies. Furthermore, the relevant information obtained from our molecular analysis emphasizes the importance of comprehensive genomic profiling for rare diseases (57). Such profiling serves as a valuable tool to better comprehend the molecular mechanisms underlying their clinical and phenotypic behaviour, and to explore potentially effective personalized treatment options.

## Data Availability

The original contributions presented in the study are included in the article/**Supplementary Material**. Further inquiries can be directed to the corresponding author.

## References

[1] Hsu HC Chen CC Huang GT Lee PH . Clonal Epstein-Barr virus associated cholangiocarcinoma with lymphoepithelioma-like component. Hum Pathol. (1996) 27:848–50. doi: 10.1016/S0046-8177(96)90460-8, PMID: 8760021

[2] Peng L Peng X Duan S Zhang Z . Epstein-Barr virus-associated lymphoepithelioma-like intrahepatic cholangiocarcinoma: A report of 3 cases and literature review. J Cent South Univ (Medical Sciences). (2024) 49:319–30., PMID: 38755729 10.11817/j.issn.1672-7347.2024.230298 PMC11103057

[3] Yang AW Pooli A Lele SM Kim IW Davies JD LaGrange CA . Lymphoepithelioma-like, a variant of urothelial carcinoma of the urinary bladder: a case report and systematic review for optimal treatment modality for disease-free survival. BMC Urol. (2017) 17:34. doi: 10.1186/s12894-017-0224-4, PMID: 28449665 PMC5408364

[4] Yun HS Lee SK Yoon G Kim HG Lee DH Na YJ . Lymphoepithelioma-like carcinoma of the uterine cervix. Obstet Gynecol Sci. (2017) 60:118–23. doi: 10.5468/ogs.2017.60.1.118, PMID: 28217683 PMC5313355

[5] Chen G Yu Q Ran H Li X Zhang T . Rare cavitary lymphoepithelioma-like carcinoma of lung: clinical experience and literature review. BMC Pulm Med. (2023) 23:246. doi: 10.1186/s12890-023-02529-x, PMID: 37407960 PMC10324186

[6] Vanoli A Di Sabatino A Biancone L Martino M Macciomei MC Zorzi F . Small bowel Epstein-Barr virus-positive lympho-epithelioma-like carcinoma in Crohn’s disease. Histopathology. (2017) 70:837–9. doi: 10.1111/his.13133, PMID: 27891660

[7] Corallo S Lasagna A Filippi B Alaimo D Tortorella A Serra F . Unlocking the potential: epstein-barr virus (EBV) in gastric cancer and future treatment prospects, a literature review. Pathogens. (2024) 13:728. doi: 10.3390/pathogens13090728, PMID: 39338919 PMC11435077

[8] Labgaa I Stueck A Ward SC . Lymphoepithelioma-like carcinoma in liver. Am J Pathol. (2017) 187:1438–44. doi: 10.1016/j.ajpath.2017.02.022, PMID: 28500863

[9] Li L Ma BBY Chan ATC Chan FKL Murray P Tao Q . Epstein-barr virus-induced epigenetic pathogenesis of viral-associated lymphoepithelioma-like carcinomas and natural killer/T-Cell Lymphomas. Pathogens. (2018) 7(3):63. doi: 10.3390/pathogens7030063, PMID: 30022006 PMC6161003

[10] Liao TC Liu CA Chiu NC Yeh YC Chiou YY . Lymphoepithelioma-like cholangiocarcinoma: A mimic of hepatocellular carcinoma on imaging features. World J Gastroenterol. (2015) 21:4089–95. doi: 10.3748/wjg.v21.i13.4089, PMID: 25852298 PMC4385560

[11] Yang Q Cai Q Wen H Mao Y Ban X Rong D . The CT and MRI features of primary intrahepatic lymphoepithelioma-like cholangiocarcinoma. Am J Roentgenology. (2021) 216:393–402. doi: 10.2214/AJR.20.22937, PMID: 33325732

[12] Ling W Lu C Huang H Qiu T Lu Q Huang C . Ultrasonographic findings of intrahepatic lymphoepithelioma-like cholangiocarcinoma associated with Epstein-Barr virus: Two cases report. Medicine. (2019) 98(3):e14206. doi: 10.1097/MD.0000000000014206, PMID: 30653176 PMC6370147

[13] Chan AWH Tong JHM Sung MYM Lai PBS To KF . Epstein-Barr virus-associated lymphoepithelioma-like cholangiocarcinoma: A rare variant of intrahepatic cholangiocarcinoma with favourable outcome. Histopathology. (2014) 65:674–83. doi: 10.1111/his.12455, PMID: 24804938

[14] Jeng YM Chen CL Hsu HC . Lymphoepithelioma-like cholangiocarcinoma: An Epstein-Barr virus-associated tumor. Am J Surg Pathology. (2001) 25:516–20. doi: 10.1097/00000478-200104000-00012, PMID: 11257627

[15] Tsai JH Liau JY Lee CH Jeng YM . Lymphoepithelioma-like intrahepatic cholangiocarcinoma is a distinct entity with frequent pTERT/TP53 mutations and comprises 2 subgroups based on epstein-barr virus infection. Am J Surg Pathol. (2021) 45:1409–18. doi: 10.1097/PAS.0000000000001716, PMID: 33859071

[16] Zhang K Tao C Tao Z Wu F An S Wu J . Lymphoepithelioma-like carcinoma in liver not associated with Epstein-Barr virus: a report of 3 cases and literature review. Diagn Pathol. (2020) 15:115. doi: 10.1186/s13000-020-01035-6, PMID: 32967689 PMC7513497

[17] Kulangara K Zhang N Corigliano E Guerrero L Waldroup S Jaiswal D . Clinical utility of the combined positive score for programmed death ligand-1 expression and the approval of pembrolizumab for treatment of gastric cancer. Arch Pathol Lab Med. (2019) 143:330–7. doi: 10.5858/arpa.2018-0043-OA, PMID: 30028179

[18] Cao J Huang L Liu C Li J Zhang X Shen J . Double primary hepatic cancer (hepatocellular carcinoma and intrahepatic cholangiocarcinoma) in a single patient: A clinicopathologic study of 35 resected cases. J Gastroenterol Hepatol. (2013) 28:1025–31. doi: 10.1111/jgh.12143, PMID: 23425127

[19] Jung KS Chun KH Choi GH Jeon HM Shin HS Park YN . Synchronous development of intrahepatic cholangiocarcinoma and hepatocellular carcinoma in different sites of the liver with chronic B-viral hepatitis: two case reports. BMC Res Notes. (2013) 6:520. doi: 10.1186/1756-0500-6-520, PMID: 24313990 PMC4029323

[20] Inaba K Suzuki S Sakaguchi T Kobayasi Y Takehara Y Miura K . Double primary liver cancer (intrahepatic cholangiocarcinoma and hepatocellular carcinoma) in a patient with hepatitis C virus-related cirrhosis. J Hepatobiliary Pancreat Surg. (2007) 14:204–9. doi: 10.1007/s00534-006-1134-0, PMID: 17384916

[21] Watanabe T . Synchronous development of HCC and CCC in the same subsegment of the liver in a patient with type C liver cirrhosis. World J Hepatol. (2009) 1:103. doi: 10.4254/wjh.v1.i1.103, PMID: 21160972 PMC2998951

[22] Jung DH Hwang S Kim KH Hong SM Lee YJ Ahn CS . Clinicopathological features and post-resection prognosis of double primary hepatocellular carcinoma and intrahepatic cholangiocarcinoma. World J Surg. (2017) 41:825–34. doi: 10.1007/s00268-016-3787-4, PMID: 27812807

[23] Liu Y Guo D He X Liu X Chen W Chen L . The MR imaging of primary intrahepatic lymphoepithelioma-like cholangiocarcinoma: A diagnostic challenge. Diagnostics (Basel). (2023) 13:2998. doi: 10.3390/diagnostics13182998, PMID: 37761365 PMC10528328

[24] Wang X Yu G Chen M Wei R Chen J Wang Z . Pattern of distant metastases in primary extrahepatic bile-duct cancer: A SEER -based study. Cancer Med. (2018) 7:5006–14. doi: 10.1002/cam4.1772, PMID: 30277653 PMC6198228

[25] Katayose Y Nakagawa K Yamamoto K Yoshida H Hayashi H Mizuma M . Lymph nodes metastasis is a risk factor for bone metastasis from extrahepatic cholangiocarcinoma. Hepatogastroenterology. (2012) 59(118):1758–60. doi: 10.5754/hge11806, PMID: 22366495

[26] Wakahara T Tsukamoto T Kitamura S Watanabe A Tsujimura T Nakamura Y . Metastatic colon cancer from intrahepatic cholangiocarcinoma. J Hepatobiliary Pancreat Surg. (2005) 12:415–8. doi: 10.1007/s00534-005-0991-2, PMID: 16258812

[27] Fujii K Goto A Yoshida Y Suzuki K Matunaga Y Shinomura Y . Gastrointestinal: Transmural colonic metastasis arising from primary cholangiocarcinoma. J Gastroenterol Hepatol. (2010) 25:1329. doi: 10.1111/j.1440-1746.2010.06396.x, PMID: 20594264

[28] Izzo F Piccirillo M Albino V Botti G Foggia M Iodice R . Case report: Appearance of an intestinal metastasis from intrahepatic cholangiocarcinoma occurring 5 years after resection of the primary tumor. Eur J Gastroenterol Hepatol. (2010) 22:892–4. doi: 10.1097/MEG.0b013e32832eb62b, PMID: 19550345

[29] Tokodai K Kawagishi N Miyagi S Takeda I Sato K Akamatsu Y . Intestinal obstruction caused by colonic metastasis from intrahepatic cholangiocarcinoma 6 years after removal of the primary tumor: Report of a case. Surg Today. (2012) 42:797–800. doi: 10.1007/s00595-012-0138-4, PMID: 22307905

[30] Vabi BW Carter J Rong R Wang M Corasanti JG Gibbs JF . Metastatic colon cancer from extrahepatic cholangiocarcinoma presenting as painless jaundice: Case report and literature review. J Gastrointest Oncol. (2016) 7:E25–30. doi: 10.3978/j.issn.2078-6891.2015.119, PMID: 27034804 PMC4783741

[31] Niazi A Saif MW . Colon mass as a secondary metastasis from cholangiocarcinoma: A diagnostic and therapeutic dilemma. Cureus. (2016) 8:e707. doi: 10.7759/cureus.707, PMID: 27588228 PMC4999351

[32] Bridgewater J Fletcher P Palmer DH Malik HZ Prasad R Mirza D . Long-term outcomes and exploratory analyses of the randomized phase III BILCAP study. J Clin Oncol. (2022) 40:2048–57. doi: 10.1200/JCO.21.02568, PMID: 35316080

[33] Oh DY He AR Bouattour M Okusaka T Qin S Chen LT . Durvalumab or placebo plus gemcitabine and cisplatin in participants with advanced biliary tract cancer (TOPAZ-1): updated overall survival from a randomised phase 3 study. Lancet Gastroenterol Hepatol. (2024) 9:694–704. doi: 10.1016/S2468-1253(24)00095-5, PMID: 38823398

[34] Ghandour F Anderson S Al-Diffalha S . Epstein-bar virus-positive lymphoepithelioma-like intrahepatic cholangiocarcinoma: A case report and literature review. Int J Surg Pathol. (2024) 33:1182–1189. doi: 10.1177/10668969241297260, PMID: 39692455

[35] Huang YH Zhang CZ yi Huang QS Yeong J Wang F Yang X . Clinicopathologic features, tumor immune microenvironment and genomic landscape of Epstein-Barr virus-associated intrahepatic cholangiocarcinoma. J Hepatol. (2021) 74:838–49. doi: 10.1016/j.jhep.2020.10.037, PMID: 33212090

[36] Sohn BH Hwang JE Jang HJ Lee HS Oh SC Shim JJ . Clinical significance of four molecular subtypes of gastric cancer identified by the cancer genome atlas project. Clin Cancer Res. (2017) 23:4441–9. doi: 10.1158/1078-0432.CCR-16-2211, PMID: 28747339 PMC5785562

[37] Chen FF Yan JJ Lai WW Jin YT Su IJ . Epstein-barr virus-associated nonsmall cell lung carcinoma. Cancer. (1998) 82:2334–42. doi: 10.1002/(SICI)1097-0142(19980615)82:12<2334::AID-CNCR6>3.0.CO;2-S 9635525

[38] Corallo S Fucà G Morano F Salati M Spallanzani A Gloghini A . Clinical behavior and treatment response of epstein-barr virus-positive metastatic gastric cancer: implications for the development of future trials. Oncologist. (2020) 25:780–6. doi: 10.1634/theoncologist.2020-0037, PMID: 32272500 PMC7485344

[39] Wang L Dong H Ni S Huang D Tan C Chang B . Programmed death-ligand 1 is upregulated in intrahepatic lymphoepithelioma-like cholangiocarcinoma. Oncotarget. (2016) 7:69749–59. doi: 10.18632/oncotarget.11949, PMID: 27626174 PMC5342512

[40] Ding Y Sun Z You W Zhang S Chang C Yan S . Lymphoepithelioma-like intrahepatic cholangiocarcinoma with Epstein-Barr virus infection: report of a rare case. Ann Transl Med. (2019) 7:497–7. doi: 10.21037/atm.2019.08.105, PMID: 31700933 PMC6803244

[41] Nogami A Saito S Hasegawa H Yoneda M Harada K Fujikawa H . Lymphoepithelioma-like cholangiocarcinoma with Epstein–Barr virus infection treated by radiofrequency ablation. Clin J Gastroenterol. (2021) 14:638–44. doi: 10.1007/s12328-020-01303-4, PMID: 33400192

[42] Zheng L Zhou N Yang X Wei Y Cheng Y Gou H . Clinicopathological features of a rare cancer: Intrahepatic lymphoepithelioma-like cholangiocarcinoma with Epstein-Barr virus infection. Clin Res Hepatol Gastroenterol. (2023) 47:102244. doi: 10.1016/j.clinre.2023.102244, PMID: 37944749

[43] Li H Xie S Liang S Pan Y Lin W Cheng N . Clinicopathological analysis of lymphoepithelioma-like intrahepatic cholangiocarcinoma. Pathol Res Pract. (2025) 268:155848. doi: 10.1016/j.prp.2025.155848, PMID: 40020332

[44] Li X Ji H Zhang D Jin M Guo X Gao P . Lymphoepithelioma-like cholangiocarcinoma with hepatitis C virus infection treated by microwave ablation: A literature review and case report. Cancer Manag Res. (2022) 14:2155–60. doi: 10.2147/CMAR.S366419, PMID: 35813580 PMC9266673

[45] Chan AWH Zhang Z Chong CCN Tin EKY Chow C Wong N . Genomic landscape of lymphoepithelioma-like hepatocellular carcinoma. J Pathology. (2019) 249:166–72. doi: 10.1002/path.5313, PMID: 31168847

[46] Fang W Zhang J Hong S Zhan J Chen N Qin T . EBV-driven LMP1 and IFN-γ up-regulate PD-L1 in nasopharyngeal carcinoma: Implications for oncotargeted therapy. Oncotarget. (2014) 5:12189–202. doi: 10.18632/oncotarget.2608, PMID: 25361008 PMC4322961

[47] Green MR Rodig S Juszczynski P Ouyang J Sinha P O’Donnell E . Constitutive AP-1 activity and EBV infection induce PD-L1 in Hodgkin lymphomas and posttransplant lymphoproliferative disorders: implications for targeted therapy. Clin Cancer Res. (2012) 18:1611–8. doi: 10.1158/1078-0432.CCR-11-1942, PMID: 22271878 PMC3321508

[48] Cancer Genome Atlas Research Network . Comprehensive molecular characterization of gastric adenocarcinoma. Nature. (2014) 513:202–9. doi: 10.1038/nature13480, PMID: 25079317 PMC4170219

[49] Qiu ZX Zhou P Wang K . Primary pulmonary lymphoepithelioma-like carcinoma response favorably to nivolumab: A case report. Onco Targets Ther. (2019) 12:8595–600. doi: 10.2147/OTT.S219512, PMID: 31802895 PMC6802557

[50] Kim C Rajan A DeBrito PA Giaccone G . Metastatic lymphoepithelioma-like carcinoma of the lung treated with nivolumab: a case report and focused review of literature. Transl Lung Cancer Res. (2016) 5:720–6. doi: 10.21037/tlcr.2016.11.06, PMID: 28149767 PMC5233874

[51] Narayanan A Knollmann FD Walby JAS Lim S Gandara DR Riess JW . EBV-positive primary pulmonary lymphoepithelioma-like carcinoma response to PD-L1 blockade. Clin Lung Cancer. (2019) 20:e238–41. doi: 10.1016/j.cllc.2018.12.015, PMID: 30679078

[52] Wu Z Xian X Wang K Cheng D Li W Chen B . Immune checkpoint blockade therapy may be a feasible option for primary pulmonary lymphoepithelioma-like carcinoma. Front Oncol. (2021) 11:626566. doi: 10.3389/fonc.2021.626566, PMID: 33981599 PMC8110193

[53] Zhu Y Dang Z Xu H Yuan Y Chen Y Li Z . High PD-L1 level of advanced hepatic lymphoepithelioma-like carcinoma response favorably to lenvatinib plus toripalimab. Cancer Sci. (2022) 113:1880–4. doi: 10.1111/cas.15339, PMID: 35298067 PMC9128174

[54] Sam Saji A Yang B Hou WT Liu X Ren QP Wei YF . Combined NK-CIK and PD-1 inhibitor (nivolumab), an effective immunotherapy for treating intrahepatic lymphoepithelioma-like cholangiocarcinoma unassociated with EBV infection: Two case reports and a literature review. Front Oncol. (2023) 13. doi: 10.3389/fonc.2023.1090580, PMID: 36865802 PMC9971717

[55] Chiang NJ Hou YC Tan KT Tsai HW Lin YJ Yeh YC . The immune microenvironment features and response to immunotherapy in EBV-associated lymphoepithelioma-like cholangiocarcinoma. Hepatol Int. (2022) 16:1137–49. doi: 10.1007/s12072-022-10346-3, PMID: 35780451

[56] Li R Cheng K Li X Chang C Lv W Xiaoying L . Case report: Immunotherapy plus chemotherapy and stereotactic ablative radiotherapy (ICSABR): a novel treatment combination for Epstein-Barr virus-associated lymphoepithelioma-like intrahepatic cholangiocarcinoma. Front Pharmacol. (2023) 14. doi: 10.3389/fphar.2023.1147449, PMID: 37614316 PMC10443589

[57] Normanno N de Luca A Abate RE Morabito A Milella M Tabbò F . Current practice of genomic profiling of patients with advanced solid tumours in Italy: the Italian Register of Actionable Mutations (RATIONAL) study. Eur J Cancer. (2023) 187:174–84. doi: 10.1016/j.ejca.2023.03.027, PMID: 37167765

