# Supplementary material for: Case Report: Epstein-Barr virus negative lymphoepithelioma-like cholangiocarcinoma: a rare tumor that deserves further exploration. Report of a case with distinct genomic and clinical features
Source: Front Oncol. 2025 Aug 4;15:1620328. doi: 10.3389/fonc.2025.1620328 (PMC12358292; doi:10.3389/fonc.2025.1620328)
Supplement: Supplementary file 1 [file DataSheet1.docx]

# Supplementary Figure 1: Immunohistochemical staining of PD-L1 expression of a tumor sample of our case showing high PD-L1 expression.

#
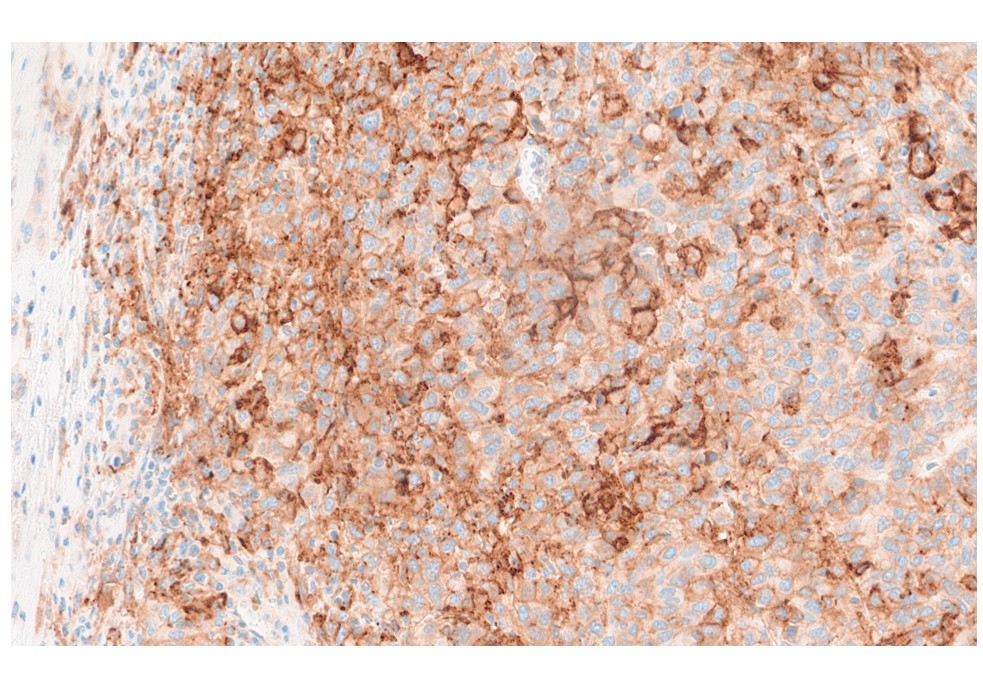


# **Supplementary Table 1**. Previous reported cases of intracolonic metastases from cholangiocarcinoma

| **Reference** | **Sex** | **Age (years)** | **Tumor Stage*** | **Liver Surgery** | **CT after liver surgery** | **Disease-free interval to colonic metastases** | **Surgery for intracolonic metastasis** | **Follow-up after itracolonic metastasis** | **Outcome** |
| --- | --- | --- | --- | --- | --- | --- | --- | --- | --- |
| Wakahara (26) | M | 62 | Metastatic | Right sectorectomy | No | Syncronous | Sdt | 12 months | AWOD |
| Fujii  (27) | W | 59 | Metastatic | No | NA | Syncronous | NR | NR | NR |
| Izzo  (28) | M | 76 | pT3N0M0 | Bisegmentectomy of V and IV segments plus extended regional lymphadenectomy | No | 5 years | Lht | NR | AWOD |
| Tokoda (29) | M | 57 | pT1N0M0 | Resection of the left lobe, Spiegel lobe, and extrahepatic bile duct | No | 6 years | Rht | 8 months | AWOD |
| Vabi  (30) | F | 61 | Metastatic | No | No | Syncronous | No | NR | DOD |
| Niazi  (31) | M | 70 | pT1N1MX | Wipple procedure | Yes^§^ | 9 months | No | 4 months | AWD |

AWD: alive with disease; AWOD: alive without disease; CT: chemotherapy; DOD: dead of disease; F: female; Lht: left hemicolectomy; M: male; NA: not applicable; NR: not reported; Rht: right hemicolectomy. Sdt: Sigmoidectomy.

*According to the 8th edition of the American Joint Committee on Cancer (AJCC) staging system.

^§^Gemcitabine (1000 mg/mq dd 1,8, q28) for 6 months

# Supplementary Figure 2: Timeline chart describing patient course.


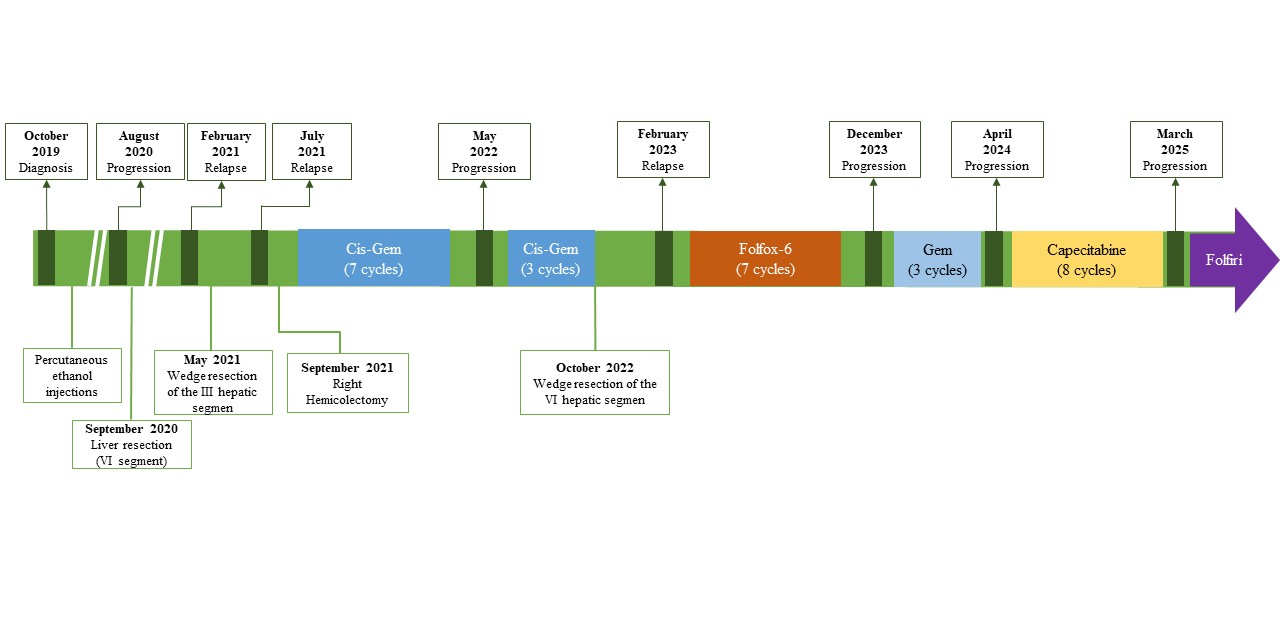


Cis-Gem: Cisplatin plus Gemcitabine
